# Behavioral Dynamics in Swimming: The Appropriate Use of Inertial Measurement Units

**DOI:** 10.3389/fpsyg.2017.00383

**Published:** 2017-03-14

**Authors:** Brice Guignard, Annie Rouard, Didier Chollet, Ludovic Seifert

**Affiliations:** ^1^Centre d’Etudes des Transformations des Activités Physiques et Sportives (CETAPS EA3832), Faculty of Sport Sciences, University of Rouen Normandy, Mont Saint AignanFrance; ^2^Laboratoire Interuniversitaire de Biologie de la Motricité (LIBM), Department Sciences and Mountain (SceM), University Savoie Mont Blanc, Le Bourget-du-LacFrance

**Keywords:** human swimming behavior, coordination variability, behavioral adaptability, inertial measurement units, aquatic environment, swimming monitoring

## Abstract

Motor control in swimming can be analyzed using low- and high-order parameters of behavior. Low-order parameters generally refer to the superficial aspects of movement (i.e., position, velocity, acceleration), whereas high-order parameters capture the dynamics of movement coordination. To assess human aquatic behavior, both types have usually been investigated with multi-camera systems, as they offer high three-dimensional spatial accuracy. Research in ecological dynamics has shown that movement system variability can be viewed as a functional property of skilled performers, helping them adapt their movements to the surrounding constraints. Yet to determine the variability of swimming behavior, a large number of stroke cycles (i.e., inter-cyclic variability) has to be analyzed, which is impossible with camera-based systems as they simply record behaviors over restricted volumes of water. Inertial measurement units (IMUs) were designed to explore the parameters and variability of coordination dynamics. These light, transportable and easy-to-use devices offer new perspectives for swimming research because they can record low- to high-order behavioral parameters over long periods. We first review how the low-order behavioral parameters (i.e., speed, stroke length, stroke rate) of human aquatic locomotion and their variability can be assessed using IMUs. We then review the way high-order parameters are assessed and the adaptive role of movement and coordination variability in swimming. We give special focus to the circumstances in which determining the variability between stroke cycles provides insight into how behavior oscillates between stable and flexible states to functionally respond to environmental and task constraints. The last section of the review is dedicated to practical recommendations for coaches on using IMUs to monitor swimming performance. We therefore highlight the need for rigor in dealing with these sensors appropriately in water. We explain the fundamental and mandatory steps to follow for accurate results with IMUs, from data acquisition (e.g., waterproofing procedures) to interpretation (e.g., drift correction).

## Introduction

Research on human swimming has been extensive in part because of one of the unique properties of water: its high density, which causes great resistance to movement. Many of the studies have been in the fields of physiology (Berger et al., 1997; Pendergast et al., 2003), biomechanics (Payton and Bartlett, 1995; Nikodelis et al., 2005; Gourgoulis et al., 2008), and motor control (Chollet and Seifert, 2011), and have helped coaches to monitor and manage training sessions. Motor control investigations follow the principles of coordination dynamics within the theoretical framework of ecological dynamics (Seifert et al., 2013; Davids et al., 2015), an approach used to study the continuous interactions between an individual and his/her environment. Applied to swimming, this framework takes the continuous swimmer–aquatic environment interaction as the most relevant scale of analysis for understanding human behavior in an ecological context of performance (Seifert et al., 2014a). According to Wei et al. (2014, p. 547), “nowhere in sport is performance so dependent on the interaction of the athlete with the surrounding medium than in competitive swimming.” In this sense, swimming provides a valuable and interesting vehicle for studying emergent behaviors from a coordination dynamics and ecological point of view. In his ecological theory of *direct perception*, Gibson (1979) argued that animals (i.e., human swimmers) perceive and act on substances (e.g., water), surfaces (e.g., swimming block), places (e.g., a swimming pool), objects (e.g., paddles) and events (e.g., a 400-m front crawl competition) in the environment, without integrating representations of the world to perceive it (Araújo et al., 2006). This theory suggests that perception guides an athlete’s actions and, in turn, his/her actions shape on-going perceptions (i.e., leading to a coupling of perception and action to support performance behaviors; Davids et al., 2015). In competitive swimming, swimmers’ actions impact the motion of water particles in a circular and tight manner since fluid motion will, in turn, impact the swimmer’s future perceptions and actions. The circular causality between perception and action, and therefore the emergence of functional behaviors, is continuously shaped by three categories of constraints: organismic (i.e., the individual characteristics of a performer), environmental (i.e., external physical and social constraints surrounding a performer), and task (i.e., the specific goals of an activity) (Newell, 1986). These constraints continually reduce the number of configurations that a complex adaptive system can adopt in a performance environment (Glazier and Davids, 2009; Davids et al., 2013). Consequently, appropriate manipulations of these constraints may prepare the swimmer to functionally respond to the competitive context of performance through adaptive behavior (Seifert et al., 2013). Adaptability refers to the subtle blend of behavioral stability and flexibility, in the sense that stability is the robustness of behavior under conditions of perturbation (e.g., waves) and flexibility is the superficial refinement of behaviors to adjust to constraints (e.g., approaching the wall to turn) (Seifert et al., 2014e). Because movement behavior emerges from the surrounding constraints that an individual must continuously cope with, we need to understand the mechanisms underlying behavioral stability, loss of stability and flexibility. Such changes in coordination dynamics are strongly dependent on the *magnitude* of the perturbation from constraints on the individual–environment system and may be related to *low-* and *high*-order parameters of behavior. Low-order parameters are generally related to common biomechanical parameters (e.g., positions, velocities, accelerations), reflecting simple inherent mechanisms (i.e., over space or time) that lead to the emergence of behavior (Haddad et al., 2006). This first level of analysis should be complemented, however, by capturing the true dynamics of the task, as doing so provides a better characterization of the rich complexity of the system (Haddad et al., 2006). High-order parameters combine multiple lower-order components, like, for example, *position* and *velocity* to obtain the relative phase between limbs, which can be used to capture the system coordination dynamics.

In swimming, low-order behavioral parameters are generally measured through two-dimensional video analyses. This method has become the gold standard (e.g., Nikodelis et al., 2005; Sanders et al., 2006; Elipot et al., 2009; Naemi et al., 2010; Mason and Formosa, 2011; Callaway, 2015) to collect kinematic data (i.e., prerequisite data for assessing behavior). First, two-dimensional analyses were “designed to identify where, why and how swimmers performed better than others” (Mason and Formosa, 2011, p. 413). The temporal parameters of events (i.e., duration of start, turn and finish segments) or stroke length (SL; i.e., distance traveled by the body during a complete stroke), stroke rate (SR; i.e., number of stroke cycles per minute), and mean stroke velocity are assessed by a digitization procedure using two-dimensional camera-based analysis. It should be noted, however, that simple manual digitization of anatomical landmarks is error-prone and the data processing is long (Wilson et al., 1999; Mooney et al., 2015a) (27 h to digitize four stroke cycles, according to Psycharakis and Sanders, 2008). In addition, Dadashi et al. (2012, p. 12928) have stated, “the biomechanical analysis of swimming remains inadequately explored due to complications of kinematic measurements in water,” leading to an increase in error reconstruction up to 42% compared with similar on-land analyses (Silvatti et al., 2013). The parallax effect at the water–air interface (Kwon, 1999), water clarity and light reflection, distortion problems and pixel contrast between the swimmer and background (Ichikawa et al., 1998), and turbulence or bubble formation (Mooney et al., 2015a) are all factors that hamper continuity in the recorded data. Despite these difficulties, however, interesting data have emerged on the spatial or temporal characteristics of the swimming path (Callaway et al., 2009), swimmers’ mechanical energy (Berger et al., 1997; Pendergast et al., 2003), and hand force production (Schleihauf, 1979; Toussaint and Beek, 1992). Yet these analyses remain limited for evaluating *higher-order* parameters, which require another level of investigation (Callaway et al., 2009; de Magalhães et al., 2014). For this purpose, researchers turned to three-dimensional optoelectronic analyses (Chiari et al., 2005) based on the automatic detection of reflective markers positioned on swimmers’ joints to properly track their motion (Callaway et al., 2009; Dadashi et al., 2013c). For an example in breaststroke, consider the real-time data collected in a calibrated volume by Olstad et al. (2012). The camera setup, position, resolution and calibration determine a *volume* within which movement will be analyzed: the more cameras used and the closer the calibration volume, the greater the measurement accuracy will be (de Jesus et al., 2015). This method is the gold standard in laboratory conditions, but remains relatively rare outdoors or in constrained environments, such as underwater (Silvatti et al., 2012; de Jesus et al., 2015). Another major issue in swimming is that the analyses are performed over a restricted area (Ceccon et al., 2013) of only three or four stroke cycles (Dadashi et al., 2013c; Callaway, 2015). This means that, although multi-camera systems can be used for inter-individual or intra-cyclic analyses of high-order movement parameters, they are of limited use for investigating behavioral dynamics.

The limitations inherent to both two- and three-dimensional video-based technologies have prompted investigators to look for new ways (de Magalhães et al., 2014) to dynamically monitor swimming training and better investigate human behavioral adaptations to surrounding swimming constraints. The *accelerometer-based data logger* (Silvatti et al., 2012; Callaway, 2015) may be one of the first devices to respond to the requirements of research in coordination dynamics. By incorporating a gyroscope (to measure angular velocities) and a magnetometer (sensitive to magnetization; for a technical complement see Dadashi, 2013; Barber, 2014; Vannozzi, 2014), a wider range of measurement opportunities is offered. This device is also called an inertial measurement unit (IMU), a wearable motion sensor (WMS) or a microelectromechanical system (MEMS) (Callaway et al., 2009), but we will refer to it as an IMU in this review. IMUs were recently validated for studying the “readily observable factors” (Glazier et al., 2006, p. 61; i.e., swimming speed, stroke length, stroke frequency) of swimming performance during *training* sessions, although equipping swimmers with sensors during competition is unauthorized (Mooney et al., 2015b). Once they are scrupulously waterproofed, IMUs offer distinct advantages to investigate swimmers’ behavior dynamics (Dadashi et al., 2013e): first, they can record a high volume (e.g., 5981 cycles recorded by Dadashi et al., 2016) of continuous data over an entire swimming training event. Second, they do not require digitization procedures (de Magalhães et al., 2014), and they are user-centric (i.e., no interference between two swimmers wearing them), low cost, and portable for easy use in field conditions (Favre et al., 2006; Dadashi et al., 2013c, 2015). Not least, the results are rapidly available for simple analyses (Dadashi et al., 2013c) once complete data processing has been performed one time (i.e., data processing depend on the quantity and complexity of the investigated parameters).

Inertial measurement units thus open new perspectives on coordination dynamics by enabling the investigation of inter-cyclic *variability* in performance, movement and coordination patterns (i.e., variability of both low- and high-order parameters). The data can then be used to build swimmer profiles and to more deeply explore swimmers’ *adaptability* to the constraints surrounding them (Newell, 1986). Seifert et al. (2014a), for example, demonstrated that there is no single and ideal pattern of coordination in swimming. Instead, these authors showed that the coordination variability observed in neurobiological systems is essential to produce (i) new behaviors that are highly adapted to the situations that arise, (ii) stable behavior despite external disturbances, and (iii) flexible behavior as a function of the constraints that continuously surround individuals (Bartlett et al., 2007; Davids and Glazier, 2010). The analysis of coordination dynamics and its functional variability provides insight into the processes by which swimmers adapt to the continuous changes in the constraining and unpredictable water environment (Bartlett et al., 2007).

In this critical review, we examine two key aspects of swimming research: (i) the characterization of human behavior in the highly resistive aquatic environment (e.g., behavior emergence, stability or flexibility) and (ii) the evaluation of inter-cyclic variability as a way to gain insight into the *coordination dynamics* of swimming behavior. Such investigations are facilitated by focusing on both low- and high-order movement parameters in order to precisely reveal the rich complexity of the swimmer–aquatic environment system. Yet although video-based analyses have been quite popular in swimming research, they do not offer the possibility of characterizing these parameters over long periods, and some researchers have thus turned to IMUs as a solution. Therefore, this critical review presents how IMUs can be used to characterize swimming behavior, providing valuable insights for both researchers and coaches. IMUs have undergone rapid development, with steadily increasing use in swimming studies (e.g., 87 references included in the review of Mooney et al., 2015b, with 62 published since 2010). To deal with this expanded literature, the present review goes beyond the technical researches performed by de Magalhães et al. (2014) and Mooney et al. (2015b), relating the research question driving each analysis to the appropriate *signal processing* from studies that have used accelerometers and IMUs. In the first section, we review the IMU-based investigations of low-order behavioral parameters and their variability. The second and main section is devoted to the assessment of *high-order* behavioral parameters (i.e., movement coordination) in swimming and inter-cycle variability. IMUs are particularly well suited to explore the functional role of variability (especially between cycles) through numerous and long time-series analyses performed in an ecological context of performance. As assessing these parameters in swimming depends greatly on sensor use in the constraining aquatic environment, the last section provides practical recommendations ranging from sensor positioning to data processing.

Journal and conference articles referencing the assessment of coordination dynamics in swimming with accelerometers or IMUs were selected from the major scientific databases: PubMed, Science Direct, IEEE Xplore, Scopus and Google Scholar. The searching keywords were “human behavior in swimming” or “swimming biomechanics” investigations for “performance” or “motion/movement analysis” or “stroke analysis” or “angle determination” or “coordination dynamics” or “sensorimotor control” purposes, analyzed with “IMUs” or “WMS” or “MEMS” or “inertial sensors” or “accelerometers” or “gyroscopes.” The inclusion criterion was the relevance of the article/conference proceeding to assessing low- to high-order parameters of human swimming behavior with the use of accelerometers, and/or gyroscopes, and/or magnetometers. Fifty articles and conference proceedings (published before 2016) were identified for review. Eleven other papers on terrestrial cyclical activities were also included for their relevance to determining sensor position and assessing joint angles in swimming. Finally, 23 other articles on the functional and adaptive roles of performance and movement variability were included for their relevance to examining within- and between-cycle and inter-individual variability. These additional references demonstrate that measurements that were generally limited to on-land conditions in the past (e.g., building dynamical biomechanical and motor control models of swimming) are now available in the aquatic environment thanks to advances in IMU technology.

## Determining Low-Order Parameters: A First Level of Analysis to Characterize Human Behavior in Swimming

One of the first and simplest ways to characterize human swimming behavior is to analyze the circumstances in which one swimmer performs better than another despite similar environmental constraints. These parameters, generally referred to as the simple mechanisms of swimming motion—or more broadly, performance-related parameters—are swimming speed, SR, SL and even segment positions throughout the stroke cycle.

**FIGURE 1:**
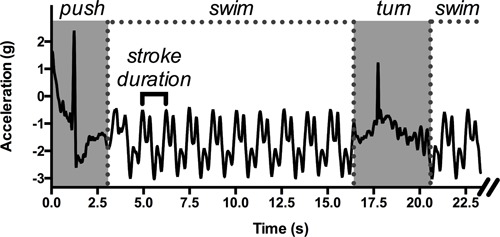
**An example of raw acceleration data (longitudinal axis) obtained with a sensor positioned on the swimmer’s lower back during a 50-m front crawl performed in a 25-m swimming pool.** Simple parameters can be identified: the duration of the wall start and the tumble turn (in gray), the swimming sequences, and the duration of one stroke cycle, allowing the computation of the average velocity and the stroke length.

Race components and stroking parameters have traditionally been assessed with stopwatches, despite inconsistencies due to athlete bias or human error (Beanland et al., 2013) and the limited number of athletes that coaches can follow at a given time (Lecoutere and Puers, 2014). The raw data from accelerometers provide the swimming time, which is the major component in swimming performance (Mooney et al., 2015b). To compute it, the beginning and end of the swimming event are recorded. Generally, the start of a swimming event is characterized by high acceleration along the longitudinal axis (Davey et al., 2008; Bächlin and Tröster, 2011; Stamm et al., 2011; Ohgi et al., 2014), decreasing to values close to zero at the end of the event. The average velocity can also be computed if the event duration and the distance covered by the swimmer are known (Bächlin and Tröster, 2011; Stamm et al., 2011; Beanland et al., 2013). This first level of analysis, which is a simple examination of the raw data when the accelerometer-based information is known (e.g., sampling frequency, reference axes), provides rapid and useful information on swimming performance. In a further step to characterize swimmers’ accelerations for a given distance, scientists can focus on the signal within the starting and ending bounds of the swimming event. In this portion of the acquired signal, the cyclical nature of the swimming activity is quite useful for determining parameters like SR, which is the time between similar acceleration peaks in the data (Ichikawa et al., 2003; James et al., 2004; Daukantas et al., 2008; Davey et al., 2008; Slawson et al., 2008; Bächlin and Tröster, 2011; Hagem et al., 2013; Khoo et al., 2013; Lecoutere and Puers, 2014; Callaway, 2015; Stamm and Thiel, 2015). SR can easily be obtained by positioning a sensor on the back or at the sacral level (**Figure 1**) (Pansiot et al., 2010; de Magalhães et al., 2014). The ratio of SR to the average velocity of the swimming lap can then give approximate values of SL and stroke index (SI, the product of velocity and SL, according to Costill et al., 1985). Additionally, in order to characterize the dynamics of performance-related parameters, scientists may now use IMUs to register data over *long periods*. The recent data loggers, which are small and able to collect data autonomously, can record up to 200 h at a sampling frequency of 100 Hz (James et al., 2011) and up to 8 h between two consecutive battery charges. This allows for a wide range of measurement contexts, from a normal training session to a complete day of data acquisition. These sensors therefore provide information on the variability in performance-related parameters—that is, for a detailed degree of swimming monitoring—that was not possible with classical video-based methods. For example, by manipulating swimming velocity or inducing fatigue, swimmers can be prompted to adapt their SR and/or SL, which can then be analyzed dynamically with IMUs. These investigations at the scale of the swimming event can be completed by the assessment of the data on temporal and/or spatial *characteristics* over restricted portions of the swimming event (e.g., a stroke cycle, start or turn sequences), which provide evidence of finer behavioral adaptations as a function of surrounding constraints.

The use, positioning and number of sensors selected for these analyses are highly dependent on the research question the scientists intend to address. Competitive swimming is aquatic locomotion involving motions of both the upper and lower limbs. Hand dynamics might be determined by a single sensor positioned on the dorsal side to detect water entry. As highlighted by Mooney et al. (2015b), detection of hand entry is *highly dependent on the swimmer’s technique*: a flatter hand entry is associated with high palmar-dorsal acceleration (Ohgi et al., 2000) or sagittal acceleration near zero (Ichikawa et al., 2003), whereas hand entry with a sharper pitch angle is associated with palmar-dorsal acceleration near 0 m/s^2^ (Ohgi et al., 2000). To investigate the dynamics of the lower limbs during training, a similar procedure is adopted, with sensors generally placed on the calf of the dominant leg (Fulton et al., 2009a,b, 2011). These results can be considered as a first level of analysis in swimming investigations, in that the movement indications are available from the *raw sensor data*. Determining the upper and lower limb oscillations offers insights into the strategies that swimmers use to create propulsion (it is generally considered that the upper limbs create nearly 90% of the total body propulsion; Deschodt et al., 1999), which can be approached by measuring their instantaneous velocity. To compute this velocity, which is considered the best parameter for estimating swimming performance (Barbosa et al., 2011; Dadashi et al., 2015), a supplementary level of analysis is needed, since velocity is not directly obtained from accelerometer or IMUs recordings. To our knowledge, Holmér (1978) was the first to assess this parameter from a single accelerometer positioned on the swimmer’s lower back. With simple instantaneous acceleration data integration, he obtained velocity curves for front crawl and breaststroke, and this pioneering investigation prompted the recent studies focused on instantaneous hip (Puel et al., 2014) and whole body (Dadashi et al., 2012, 2013a,b,d; Stamm et al., 2013a,b) velocity estimation following the same computational process. However, this transformation is sometimes subject to drift and needs additional processing to correct it (Dadashi et al., 2015), as described in the practical implications section. Dadashi et al. (2012, 2014) assumed that the average trend of instantaneous velocity peaks is quasi-constant due to the steady regime of front crawl swimming. They therefore extracted the cycle minimum and maximum peaks of instantaneous velocity and fit them with a shape preserving spline (Fritsch and Carlson, 1980). Then, they assessed the instantaneous velocity by expressing the data into an external reference frame (e.g., as a function of the gravity vector) to obtain a general overview of the swimmer’s velocity profile. Thus, only the component collinear with the swimmer’s displacement axis was needed to estimate swimming performance (Barbosa et al., 2011; Dadashi et al., 2015). In contrast, Stamm et al. (2013a,b) did not correct the integration error during the instantaneous velocity estimation of a push-off and swimming lap, considering noise as insignificant for such brief events. Their results showed an acceptable difference (bias of -0.15 m/s) between the values obtained with the IMU and the gold standard (i.e., tethered optical velocity meter: description in Davey and James, 2008) for both push-off and swimming lap instantaneous velocity. Some authors were even able to characterize the variability in instantaneous swimming velocity over different time-scales, showing that swimmers functionally adapt to surrounding constraints (Newell, 1986). To do so, they computed the so-called *intra-cyclic velocity variations* (IVV; Dadashi et al., 2013a) and *cycle velocity variation* (Dadashi et al., 2016) from the values of instantaneous velocity.

All the aforementioned parameters, which are listed in **Table 1**, offer a first level of analysis for investigating human swimming behavior, essentially based on temporal parameters (acceleration and its first integration). These analyses can be completed by investigations focusing on the spatial parameters of the swimming stroke.

**Table 1:** Studies focusing on the temporal low-order parameters of swimming behavior.

Authors	Measured parameters	Sensor type	Participant
Bächlin and Tröster, 2011	Wall push-off, end of the laps, average velocity, SL and SR	3D accelerometer	18 swimmers
Beanland et al., 2013	Stroke count, mid-pool velocity	3D accelerometer	21 swimmers
Callaway, 2015	Lap time, average velocity, stroke count, stroke duration, SR	3D accelerometer	12 swimmers
Dadashi et al., 2012, 2014	Instantaneous swimming velocity	3D accelerometer, 3D gyroscope	20 and 8 swimmers
Dadashi et al., 2013a	Instantaneous swimming velocity, intra-cyclic velocity variations	3D accelerometer, 3D gyroscope	12 swimmers
Dadashi et al., 2013d	Instantaneous swimming velocity, cycle mean velocity	3D accelerometer, 3D gyroscope	20 swimmers
Dadashi et al., 2015	Breaststroke cycle mean velocity	3D accelerometer, 3D gyroscope	15 swimmers
Daukantas et al., 2008	Lap count, instantaneous SR	3D accelerometer	4 swimmers
Davey et al., 2008	Wall push-off, turns, lap time, stroke count and SR	3D accelerometer	6 swimmers
Fulton et al., 2009a,b	Kick count and kick rate	3D accelerometer, 1D gyroscope	14 and 12 Paralympic swimmers
Fulton et al., 2011	Kick count and kick rate	3D accelerometer, 1D gyroscope	12 Paralympic swimmers
Hagem et al., 2013	SL, SR, lap time	3D accelerometer	1 swimmer
Ichikawa et al., 2003	Stroke frequency, hand water entry and exit	3D accelerometer	4 swimmers
James et al., 2004	Wall push-off, stroke style and stroke count metrics	3D accelerometer	Selection of swimmers
Jensen et al., 2013	Rest and swimming phases, swimming style and turn detection	3D accelerometer, 3D gyroscope	12 swimmers
Khoo et al., 2013	Acceleration profiles, stroke duration, breathing pattern	3D accelerometer	2 swimmers
Lecoutere and Puers, 2014	Split times, stroke frequencies, breathing patterns and distance per stroke	3D accelerometer, 3D gyroscope	1 swimmer
Le Sage et al., 2010a	Turns, stroke duration	3D accelerometer, 2D gyroscope	1 swimmer
Le Sage et al., 2010b	Lap time, turn detection	3D accelerometer, 3D gyroscope	1 swimmer
Ohgi et al., 2014	Rest and swimming phases, start, turns, goal touch events, swimming style	3D accelerometer	45 swimmers
Pansiot et al., 2010	Swimming style, wall push-off, lap counts	3D accelerometer	1 swimmer
Puel et al., 2014	Rotational speeds and translational accelerations, hip longitudinal speeds in breaststroke and crawl	3D accelerometer, 3D gyroscope, 3D magnetometer	Sample of swimmers
Siirtola et al., 2011	Swimming style, turns, number of strokes	3D accelerometer	11 swimmers
Slawson et al., 2008	Stroke count, stroke duration	3D accelerometer	1 triathlete
Stamm et al., 2011	Start and end swimming times, stroke frequency, average velocity	3D accelerometer	1 swimmer
Stamm et al., 2013a	Instantaneous push-off and glide velocities	3D accelerometer	7 swimmers
Stamm et al., 2013b	Instantaneous swimming velocity, SR	3D accelerometer, 3D gyroscope	17 swimmers
Stamm and Thiel, 2015	Lap velocity and acceleration, SR, arm symmetry	3D accelerometer, 3D gyroscope	8 swimmers
Vannozzi et al., 2010	Gliding phase, stroke phase and turn phase durations	3D accelerometer, 2D gyroscope	8 swimmers

*For each work, the measured parameters are included alongside the sensor type. SL, stroke length; SR, stroke rate; 1D, one-dimensional; 2D, two-dimensional; 3D three-dimensional.*

Kinematic analyses provide clear insight into swimmers’ continuous functional adaptations to the changes in their dynamic and unpredictable aquatic environment. Indeed, by determining the different limb positions in the swimming event (i.e., movement phases) and connecting them to propulsion, researchers can show how swimmers act on their environment to create or at least maintain their instantaneous velocity. Determining instantaneous velocity *variations* as a function of limb position is accomplished by coupling accelerometers or IMUs with video-analyses. For example, phases of the start (Chakravorti et al., 2013), turn (Vannozzi et al., 2010; Lee et al., 2011b; Le Sage et al., 2012; Slawson et al., 2012; Stamm et al., 2013a) (the procedures are summarized by Mooney et al., 2015b) and stroke cycle (Ohgi et al., 2000, 2003, 2014; Ohgi, 2002; Nakashima et al., 2010; James et al., 2011; Lee et al., 2011a; Callaway, 2015) have been detected, based on acceleration data. Ohgi et al. (2000, 2003) were the first to present a case study of the movement phases in the stroke cycle in both freestyle and breaststroke swimming from a single two-dimensional accelerometer. In the breaststroke and butterfly, propulsive phases are systematically followed by non-propulsive phases, as the two upper and two lower limbs make similar and simultaneous movements, causing considerable velocity fluctuations throughout the stroke cycle. For example, velocity during the breaststroke glide decreases greatly in preparation for the strong re-acceleration in the following propulsive phases of the stroke. It is thus essential to determine limb position to ensure that leg propulsion is not concomitant to upper limb propulsion, which is generally a beginner’s error (i.e., “accordion” propulsion mode; Leblanc et al., 2009). In freestyle, these fluctuations are subtler, since propulsion is created by the continuous actions of both upper and lower limbs. In the pioneering study of Ohgi et al. (2000), the authors positioned a sensor on the wrist to detect acceleration peaks, in line with the stroke phases described by Maglischo (1982), and recorded swimmers’ actions with bottom- and side-view cameras. The changes in the acceleration profile were linked to changes in forearm position, thereby defining the different stroke phases. The minimum values of sagittal hand acceleration were observed when the hand entered the water. Similarly, the maximum acceleration values were noted after the catch point at the beginning of the insweep movement. On the longitudinal axis, the values decreased to nearly zero at the beginning of the underwater sequence, since hand acceleration was greatly reduced along this axis during the entry and stretch (i.e., extension of the arm forward). High acceleration was then recorded at the beginning of the downsweep. Following this sequence, the upsweep started with vertical hand acceleration. The authors concluded that a single sensor positioned on the wrist was sufficient to detect most of the swimmer’s upper limb swimming phases (except the hand release from the water), which greatly reduced the processing time generally associated with similar video analyses (Ohgi et al., 2000). Propulsion was mainly accomplished by the completion of an efficient insweep movement, where the highest acceleration levels were recorded. Also, the acceleration curves offered insights into those instants when propulsion might be increased and thus provided indications to further adapt the swimming stroke. Nevertheless, this work must be viewed as the first step in automatically detecting the stroke phases of front crawl swimming, since it involved only one swimmer and an additional camera system. It also had to be personalized for the subject (Callaway, 2015) in terms of sensor fixation, location and orientation, the swimmer’s technique, and signal processing (e.g., low-pass filtering cutoff frequencies). Currently, coupling three-dimensional accelerometers and video systems is considered appropriate for investigating the motion phases of swimming training (as recently confirmed by Callaway, 2015, for front crawl phase determination). In other studies, a second data source (i.e., three-dimensional gyroscope) was added to the accelerometer-video couple to obtain angular velocities, further improving phase detection accuracy and validity. IMU data alone may indeed be too imprecise since these sensors provide only *estimations;* systematic control with video may thus be essential. For Ohgi (2002) and Lee et al. (2011a), the gyroscope complemented the accelerometer (positioned at the wrist) and provided additional data on the front crawl phases. Specifically, these author’s distinguished local maximum and minimum values for the angular velocity profiles corresponding to the entry, catch and exit points of the hand trajectory (Lee et al., 2011a). Dadashi et al. (2013b) used another method based on the hidden Markov model (HMM) to determine breaststroke phases. These authors hypothesized that the phases of arm and leg movement during the breaststroke possess statistical properties that can be used to supervise learning based on the HMM. For example, the breaststroke arm recovery takes place before gliding with arms fully extended. Such an event can be (i) detected and (ii) fully automated using the HMM, as can the other two stroke phases, the glide and propulsion, which, when connected to the swimming velocity, reveal swimmers’ propulsion strategies.

The discrimination of stroke phases has become feasible with IMUs (Daukantas et al., 2008; results presented in **Table 2**) and doing so reveals how swimmers are able to continuously act on their environment to minimize instantaneous velocity variations. Using IMUs *with* video, however, runs counter to the primary intention of using an *independent* system that does not have the inherent limitations of video systems, which record data over a restricted volume of analysis (Callaway et al., 2009). New investigations, mainly focusing on the assessment of higher-order parameters of swimming behavior, have therefore been conducted using only accelerometers and IMUs, which can record over long periods of time.

**Table 2:** Studies focusing on the spatial low-order parameters of swimming behavior.

Authors	Measured parameters	Sensor type	Participant
Callaway, 2015	Discrimination of stroke phases	3D accelerometer	12 swimmers
Chakravorti et al., 2013	Detection of glide phase, first stroke initiation and turn initiation	3D accelerometer, 2D gyroscope	2 swimmers
Dadashi et al., 2013b	Detection of breaststroke phases	3D accelerometer, 3D gyroscope	7 swimmers
James et al., 2011	Arm stroke identification	3D accelerometer, 3D gyroscope	1 swimmer
Lee et al., 2011a	Hand water entry and exit, discrimination of stroke phases	3D accelerometer, 3D gyroscope	6 swimmers
Lee et al., 2011b	Discrimination of tumble turn phases	3D accelerometer	2 swimmers
Le Sage et al., 2012	Turn phases, stroke count, stroke duration	3D accelerometer	12 swimmers
Nakashima et al., 2010	Wrist trajectory	3D accelerometer, 3D gyroscope	1 swimmer
Ohgi et al., 2000	Discrimination of stroke phases	2D accelerometer	2 swimmers
Ohgi, 2002	Discrimination of stroke phases	3D accelerometer (prototype I); 3D accelerometer, 3D gyroscope (prototype II)	2 swimmers
Ohgi et al., 2003	Discrimination of breaststroke phases	2D accelerometer	2 swimmers
Ohgi et al., 2014	Discrimination of stroke phases	3D accelerometer	45 swimmers
Slawson et al., 2012	Tumble turn phases	3D accelerometer, 2D gyroscope	1 triathlete

*For each work, the measured parameters are included alongside the sensor type. 2D, two-dimensional; 3D, three-dimensional.*

## Investigation of High-Order Parameters to Characterize Coordination Dynamics and Behavioral Variability in Swimming: New Perspectives Using IMUs

Combining spatial and temporal data (i.e., mixing low-order parameters) is one way to investigate the interaction of the components of the swimmer–aquatic environment system at a behavioral level. Computing the so-called spatial-temporal coordination between two or more segments (or joints) starts with the determination of the angular times series of the segments (or joints) under consideration (Wheat and Glazier, 2006). When these angles are determined using IMUs, the data processing should take into account drift, offset, sensor synchronization and three-dimensional position determination, all of which implies complex steps compared with the procedures for determining low-order parameters. These investigations are made possible by considering the orientation of the sensors in the tridimensional domain, without any further help from video systems. Sensor orientation is determined by fusing the data from the acceleration integration and angular velocity (assessed by gyroscopes). As noted, this operation has to deal with the problem of drift, whose negative effects can be limited by using a magnetometer coupled to the accelerometer and gyroscope. The magnetometer compensates for the baseline drift in the other two devices (Callaway et al., 2009). The studies on this problem are very recent, but already they point to new ways to better characterize the spatial-temporal coordination of human swimming behavior and its variability over time (works on this topic are summarized in **Table 3**).

**Table 3:** Studies focusing on the high-order parameters of swimming behavior.

Authors	Measured parameters	Sensor type	Participant
Dadashi et al., 2011	Propulsive phases, coordination index	3D accelerometer, 3D gyroscope	7 swimmers
Dadashi et al., 2013c	Arm stroke phases and inter-arm coordination	3D accelerometer, 3D gyroscope	7 swimmers
Dadashi et al., 2016	Intra-cyclic velocity variation, cycle velocity variation and inter-arm coordination	3D accelerometer, 3D gyroscope	18 swimmers
Seifert et al., 2014c	Inter-segmental elbow and knee angles cycle per cycle, arm-leg coordination	3D accelerometer, 3D gyroscope, 3D magnetometer	Not specified
Seifert et al., 2014d	Inter-segmental elbow and knee angles cycle per cycle, patterns of coordination	3D accelerometer, 3D gyroscope, 3D magnetometer	Not specified
Seifert et al., 2014e	Adaptability of limbs movements and arm-leg coordination after perturbation	3D accelerometer, 3D gyroscope, 3D magnetometer	6 swimmers

*For each study, the measured parameters are included alongside the sensor type. SR, stroke rate; 3D, three-dimensional.*

**FIGURE 2:**
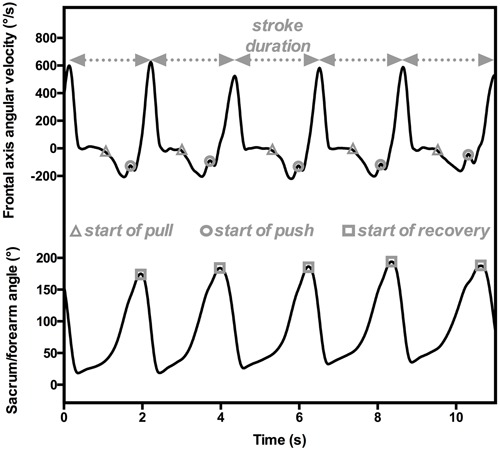
**An example of temporal parameter estimations for computing IdC from sensors positioned on the forearm and sacrum.** The angular velocities on the transversal axis (upper panel) are used to detect the beginning of pull (gray triangles) and push (gray circles), and hence the stroke duration. The absolute sacrum/forearm angle characterizes the beginning of recovery (gray squares).

Dadashi et al. (2011, 2013c) computed the inter-arm spatial-temporal coordination in front crawl swimming by assessing the Index of Coordination (IdC; Chollet et al., 2000). The IdC “measures the coordination of arm stroking, with precise quantification of the lag between the start of the propulsion by one arm and the end of propulsion by the other” (Chollet et al., 2000, p. 54). It combines inputs from both the position of the swimmer’s segments and the temporal parameters to detect each stroke phase computed using an experimental setup composed of three IMUs positioned on the swimmer’s forearms and sacrum. The IdC is computed by detecting the beginning of the pull (i.e., start of the propulsive sequence, when the forearm begins its backward motion), the beginning of the push (i.e., transition between the two propulsive phases, when the forearm passes the shoulder), and the end of the push or the beginning of the recovery (i.e., end of the propulsion, when the forearm is alongside the body) for each upper limb (Chollet et al., 2000). Dadashi et al. (2011, 2013c, 2015) automatically determined the beginning of the pull and the push by focusing on forearm angular velocities and accelerations and computing the angle between the forearms and the sacrum to assess the beginning of recovery. The beginning of the pull was characterized by high forearm velocity in the backward direction. To precisely detect this event in time, a model of slope change detection was applied for medial-lateral angular velocity and anterior-posterior acceleration curves (CUSUM algorithm; Gustafsson, 2000). The start of the push phase, which is the change in forearm movement from outsweep to insweep, was detected from the angular velocity on the frontal axis. On the curve, it was determined by the local maximum directly observable after the beginning of the pull (**Figure 2**). For the start of the recovery, these researchers had to detect the end of the underwater part of the arm motion. To do so, they focused on the absolute sacrum/forearm angle, considering the minimum angulation value when the swimmer had the arm extended to the front (i.e., during glide). Consequently, the underwater portion of the stroke corresponded to an increase in this angle until a maximum, at which point the recovery began. Due to the cyclical nature of swimming, this procedure could be performed throughout the swimming event (i.e., in dynamics), providing a complete and accurate overview of the front-crawl inter-arm coordination of the swimmers.

Another possibility for assessing coordination is to compute the continuous relative phase (CRP) between two or more segments for swimming motion analysis (Seifert et al., 2014c,d,e). This parameter contains information about both the position and velocity of the two segments under consideration. In this case, the researchers were interested in investigating inter-limb coordination, which meant accurately locating the limbs in three-dimensional space to obtain their positions, and hence the angle between them. Computing CRP was facilitated using a net of four IMUs positioned on the forearm, upper arm, thigh and shank of a body side to capture elbow and knee angles (Seifert et al., 2014c,e). The authors developed a process to correct for drift, based on the assumption that the orientation of the magnetic field would be constant during recording. This technique could not be used to compute absolute angles since the initial signal had been modified. Therefore, the authors computed *relative* angles (*𝜃_norm_*; normalized between -1, corresponding to -120°, and +1, corresponding to 50°; Seifert et al., 2014e) as the integration of the difference in the gyroscopic signals from the sensors positioned around the joint under study. A similar procedure was used to compute the normalized angular velocities (*ω_norm_*) of both elbow and knee joints in the interval [-1, +1]. Then, phase angles (*φ_elbow_* and *φ_knee_*) in degrees were calculated and corrected according to their quadrant to ensure their continuity over time (Hamill et al., 2000): *φ = arctan(ω_norm_/𝜃_norm_)*. Finally, the CRP for a complete cycle was calculated as the difference between the two phase angles (Hamill et al., 2000): *φ_rel_ = φ_elbow_–φ_knee_*. Values obtained from CRP computations directly reveal the mode of coordination between the two considered segments/joints (here knee and elbow for upper/lower limb coordination in breaststroke). In these studies (Seifert et al., 2014c,e), CRP values between -30 and +30° signified an “in-phase” pattern of coordination between knee and elbow, whereas values ranging between -180 and -150° or +150 and +180° denoted an “anti-phase” pattern.

These essential spatial-temporal investigations have for too long been limited to a restricted number of stroke cycles, but IMUs now offer the possibility of more fully considering the coordination dynamics and its variability. This objective can be achieved by characterizing the *inter-cyclic movement variability* (Dadashi et al., 2016), which can be analyzed within or between individuals. In the traditional cognitivist approach to coordination dynamics, variability in movement and coordination is noise or random fluctuation and is generally seen as detrimental to performance (Newell and Corcos, 1993; Davids et al., 2003, 2012). From this perspective, an athlete is expert when he/she is able to automate and reproduce a specific movement or coordination pattern consistent with the task goal (Ericsson et al., 1993; Abernethy et al., 2008). However, variability is inherent to all biological systems, since a repeated movement is never identical to the one before it (Bernstein, 1967). Within the ecological dynamics framework, an athlete’s movement emerges from the interaction of a set of constraints (environment, task and organism; Newell, 1986) through circular relationships between perception and action (Davids et al., 2012). The specificity of swimming is that the environmental constraint is crucial; since it limits swimmers’ motion by high resistances (water is 800 times denser than air). According to the ecological dynamics perspective, the manipulation of constraints is likely to prompt new behavior to emerge, to stabilize a given behavior, or to train adaptive flexibility. Recently, Dadashi et al. (2016) used this behavioral approach by constraining or perturbing swimmers in order to visualize the possible consequences on coordination. Such perturbations may take different forms, from the use of a parachute to add resistance, the modification of SR with a pacer positioned under the cap, or the manipulation of the fluid flow by swimming in a flume.

In the ecological dynamics framework, movement and coordination variability might be defined as functional flexibility, with the sensorimotor system adapting to continuous or temporary changes in constraints (Davids et al., 2003; Seifert et al., 2013). Variability can thus be seen as reflecting the property of *degeneracy* in the neurobiological system (Edelman and Gally, 2001; Mason, 2010; Whitacre, 2010). Degeneracy is “the ability of elements that are structurally different to perform the same function or yield the same output” (Edelman and Gally, 2001, p. 13763). In other words, different components of the sensorimotor system (i.e., *heteromorphic* components) can achieve the same task goal or performance outcome (i.e., *isofunctionality*) under specific conditions in order to ensure robustness if the initial system configuration fails (Mason, 2010). As an example, Seifert et al. (2014b) manipulated glide duration during a 200-m freestyle event and observed that swimmers were able to increase their kicking (e.g., to a 10-beat kick) to functionally adapt their behaviors when required to increase the glide phase with their arms. This functional adaptability is called *degenerate* behavior, and it should be distinguished from *redundancy*, which requires the *isomorphy* (i.e., the quality of being structurally identical; Mason, 2010) and *isofunctionality* of components to perform a function (Mason, 2010). Redundancy is characterized by a duplication of the motor program in case of failure of the initial command.

Degeneracy can be observed through different combinations of the same components (i.e., different coordination patterns) and the *interchangeability* of different structures. The concept of degeneracy therefore explains why an individual can vary sensorimotor behavior (structurally) without compromising function, providing evidence for the adaptive and functional role of movement and coordination pattern variability in order to reach a task goal. Degeneracy in the complex neurobiological systems involved in swimming, and more broadly the functional role of movement and coordination variability, can be examined at three levels: (i) inter-limb coordination variability within a cycle, (ii) variability of coordination patterns between cycles, and (iii) inter-individual variability of coordination patterns (for a review, see Seifert et al., 2014a). Seifert et al. (2014a) explained the usefulness of assessing the behavioral variability of motor coordination and control by emphasizing the effects of constraint manipulation on swimming. The investigation of degeneracy requires the computation of various indicators of variability to assess *recurrence*, *stationarity* or *cyclicity* over long time series (for a review, see Bravi et al., 2011), which might be captured with the help of IMUs. Although standard deviation, variance and coefficients of variation are often computed, more advanced analysis is useful in this case. For example, the variability between two time series can be assessed by computing the root mean square (RMS) error and the Cauchy criterion (Chen et al., 2005; Rein, 2012). The RMS measures the similarity between the pattern of one stroke cycle and the mean pattern for time continuous data, whereas the Cauchy criterion compares two consecutive coordination patterns (Chen et al., 2005; Rein, 2012). Another approach derived from unsupervised machine learning is cluster analysis to recognize patterns both between and within individuals. Applied to swimming, this approach can distinguish athletes as a function of their specialty (Figueiredo et al., 2012) or their profiles during front crawl starts (Seifert et al., 2010; Vantorre et al., 2010). In breaststroke, clustering differentiates arm to leg coordination strategies between learners of different skill levels (Seifert et al., 2011) or as a function of speed increase (Komar et al., 2015). More broadly, Dadashi et al. (2013c, 2015, 2016) provided an interesting perspective on how IMUs can be used to assess variability in swimming, and this was recently complemented by an investigation of the effects of artificial perturbation on swimmers’ behavior (Seifert et al., 2014e). These authors investigated the *flexibility* and *stability* of motor behavior by assessing the relaxation time (i.e., the time needed to recover the initial motor behavior after perturbation) (Scholz et al., 1987; Seifert et al., 2014e). To do so, swimmers performed 15 cycles at a given velocity in a flume and were then towed 1 m backward from their initial place. Following this perturbation, they had to return to their initial position as fast as possible before continuing to swim for an additional 15 cycles. Using this procedure, Seifert et al. (2014e) investigated the breaststrokers’ adaptability (ratio between stability and flexibility) in overcoming the artificial perturbation, as reflected by the inter-limb coordination variability (based on the computation of CRP). These preliminary findings obtained with IMUs must be confirmed and reinforced in the future, since this technology offers substantial advantages by capturing continuous data over long time periods (Dadashi et al., 2013c).

### Practical Implications and Technical Recommendations to Assess The Behavioral Dynamics in Swimming

Research on the low- to high-order parameters of swimming behavior has developed rapidly with the use of accelerometers and IMUs, generating new procedures from data collection to data treatment. Yet despite the undeniable possibilities offered by IMUs, scientists should bear in mind that working with IMUs in an aquatic environment is not as straightforward as it might appear. We present some recommendations ranging from sensor positioning to data processing, and we insist that any use of these tools requires a meticulous approach.

Although there is no consensus on how best to attach and seal sensors (de Magalhães et al., 2014), several suggestions have been made. First, the direction of the accelerometer and gyroscope signals depends on the device orientation. This is a crucial consideration for analyses to determine the precise orientation of IMUs in three-dimensional space (see following section), whereas it is of limited interest for studies to investigate temporal-related parameters of swimming motion. In any case, IMUs are always positioned on the skin. Different positioning procedures have been proposed but all should be meticulously followed, especially for coordination assessment. de Magalhães et al. (2014) and Mooney et al. (2015b) made note of several possibilities. To optimize placement and reduce skin movement artifacts, the relative movement between sensor and body segment should be minimized by robustly strapping or taping the sensor to the limb (Fong and Chan, 2010) to prevent it from becoming obtrusive and uncomfortable. For example, hand acceleration is easily determined by a sensor placed on the dorsal side of the hand or at the wrist. The dynamics of the leg kick can be correctly estimated by positioning a sensor on the feet or the ventral portion of the calf (for further information on sensor placement, please refer to de Magalhães et al., 2014). The sensor may be cumbersome (on average 50 × 35 × 15 mm between 2013 and 2015, according to Mooney et al., 2015b), however, and this can modify the swimming pattern and increase drag (James et al., 2011; Dadashi et al., 2013c). In one notable example, the reflective markers in motion capture analysis (with dimensions similar to IMUs) were found to increase maximal drag by 10% (Kjendlie and Olstad, 2012). These potential problems might limit the utility of IMUs over long periods of swimming more than technological factors like battery life. Finally, the sensor position must not perturb swimmers’ interactions with their environment (Bächlin and Tröster, 2011) (e.g., a sensor placed on the hand might limit sensory information from the water).

Once the sensors are placed, the first level of analysis can begin, facilitated by the cyclical nature of swimming locomotion. The raw acceleration data present a repeatable pattern (i.e., corresponding to one complete stroke), which makes it easier to determine the different portions of the event (i.e., starts and turns) (James et al., 2004; Davey et al., 2008; Le Sage et al., 2010a,b; Vannozzi et al., 2010; Bächlin and Tröster, 2011; Siirtola et al., 2011; Jensen et al., 2013; Ohgi et al., 2014). For example, the rotation during the freestyle turn is detected when an acceleration peak occurs along the transversal axis (Davey et al., 2008). In this sense, a turn is an indicator of the end of a lap or a separator of two swimming styles during a medley, and it is therefore key for measuring the time to cover a given swimming distance (Jensen et al., 2013). The data help coaches by offering many ways to monitor swimming training, since most of the sensors collect data in portable data loggers (Fong and Chan, 2010) that can be consulted at the end of a training session to provide *feedback* to the swimmers (Hagem et al., 2013; Callaway, 2015; Dadashi et al., 2015). First, the race components and temporal stroke parameters can easily be computed by focusing on the shape of the acceleration versus time curves, revealing swimmers’ inherent strategies for managing the training session and, by extension, a competitive event. Additionally, pioneering studies have investigated the status and role of variability in race management, performance optimization, training processes and skill acquisition, generally observed as a function of the manipulation of the constraints surrounding action (Newell, 1986). These constraints prompt behavioral adaptations, such as (i) increasing SR to overcome the drag created by the swimmer in the next lane, (ii) regulating swimming speed when approaching the wall to perform a turn, or (iii) showing fatigue at the end of a race (Dadashi et al., 2016). Also, the acceleration data recorded over a training session can help coaches to distinguish between swimming styles (Pansiot et al., 2010; Hou, 2012; Jensen et al., 2013; Ohgi et al., 2014; Mooney et al., 2015b), but not between two different signals emerging from the same swimming style. This means that a single sensor positioned on the chest can distinguish the signals obtained in freestyle and backstroke from those obtained in butterfly and breaststroke through the general shape of the acceleration versus time curves (Le Sage et al., 2011; Jensen et al., 2013; Ohgi et al., 2014) (please refer to Le Sage et al., 2011; Ohgi et al., 2014, for a depiction of these curves). It is therefore possible for coaches and swimmers to accurately record the swimming distances in each style during a training session and adapt exercise (e.g., swimming at high SR, with paddles or fins, or against a high fluid flow) in preparation for future competitions. When used this way, the sensors function as a “training partner” alone or coupled with other devices (e.g., heart rate monitor or VO_2_ consumption estimator) in many training contexts and over long periods of time (e.g., they may provide insight into fatigue effects after an exhausting training session; Dadashi et al., 2016). They provide data on behavioral variability in training and expand the possibilities for monitoring beyond the traditional embedded devices, like instrumented paddles (measuring force or pressure on the hand during propulsion; Chollet et al., 1988, 1992) or the Aquapacer^TM^ (positioned under the cap to maintain a target pace; Thompson et al., 2002, 2004). The feedback on swimmers’ motor behavior and performance variability is *specific* and subject-dependent (Callaway, 2015; Dadashi et al., 2015) and in this sense can help them manage their training (e.g., see Hagem et al., 2013) and, by extension, future swimming competitions. Finally, coaches can obtain an overview of their athletes’ performances by using three-dimensional accelerometers several time a year to (i) quantify the typical movements of a swimming sequence and (ii) accurately detect the temporal events in training (starts, turns, and “free swim” duration) (Chambers et al., 2015).

For the second level of data processing to assess coordination, the sensor orientation needs to be accurately captured in three-dimensional space and swimmers’ limb angles have to be estimated. This procedure generally begins with the sensor position expressed in (i) a terrestrial reference frame or (ii) a local reference frame (i.e., the limb with the sensor on it). Few studies have computed swimmers’ joint angles from IMUs, since the process to do so is both laborious and error prone. Indeed, IMUs provide *estimations* of inter-segmental angles, but they cannot compute the real joint angle since few of them store anthropometric models (or these systems have not yet been validated in aquatic environments). The first problem is deciding on the correct number of sensors and how to position them (Yang and Li, 2012) in line with the research topic. Although a single sensor positioned on the lower back allows instantaneous velocity computation (Dadashi et al., 2012, 2014), assessing upper and lower limb angles requires multiple sensors on the propulsive body segments (Seifert et al., 2014e). As the sensors have a reference frame and the recorded values are linked to the IMU positions on the subject (Seel et al., 2014), the estimation of joint angles could be affected if a sensor is not correctly aligned with the limb axes after fixation (which might be characterized by an offset; Le Sage et al., 2011), causing measurement errors. In swimming, an example of offset removal was described for body roll estimation by (i) having the subject lie face down for 10 s (zero degrees of roll was parallel to the pool deck in this position) and then (ii) adding the obtained averaged value or subtracting it from the calculated hip roll (this simple linear correction was only acceptable if the offset was considered in a single plane; Barber and Barden, 2014). This correction might be too simple to estimate the three-dimensional angle between two IMUs in water, however. Researchers should therefore follow the well-described procedure for terrestrial activities, where the offset is estimated using an immobile, known standing posture prior to its removal from the joint angle data (e.g., in walking; Mills et al., 2007; Cooper et al., 2009). Another way to correct for this error is to convert the sensor axes to the *bone anatomical frame* to obtain accurate estimations of joint angles (Chardonnens et al., 2012; Dadashi et al., 2012). Once again, the procedure has been fully described for terrestrial locomotion such as walking and running (Favre et al., 2008, 2009). Owing to a *functional calibration*, the signal synchronized between all the sensors became insensitive to their placement and was expressed in the bone anatomical frame. The procedure consisted of determining a constant rotation matrix (or a quaternion transformation) between the measured sensor frame and the segment orientation. The athletes performed a series of dynamic exercises (squats) and then maintained a standing position (for 5 s). The complete explanation of the correction process was presented by Favre et al. (2009). Moreover, this strategy can be used in water by calibrating in dry-land conditions before starting the aquatic tests, as described by Fantozzi et al. (2016) in *simulated swimming* (i.e., legs constrained and upper limbs moving in the air). The results showed good agreement between the joint angles computed with the IMUs and the gold standard (coefficients of multiple correlations near 1).

Last, Favre et al. (2006) proposed two methods to *reduce drift*, both using the motionless instants of the recording sequence (i.e., zero-velocity updates; Woyano et al., 2016). The first method relies on the quaternion-based integration of angular velocity. Orientations are expressed using vector and scalar quaternion representations from the sensor xyz reference frame into an external XYZ reference frame. The computation then proceeds by integrating the angular velocity. The second method presents an orientation correction using *gravity* (the method often used in traditional analysis: O’Donovan et al., 2007; Takeda et al., 2009; and clinical gait analysis: Cutti et al., 2010; Ferrari et al., 2010). It assumes that limb acceleration has two components at each instant: one due to gravity, the other to the motion of the sensor. During rest (or near constant motion velocity), the accelerometer measures only the effects of gravity. *Static* calibration takes advantage of gravity, being the signal common to all IMUs. Then, an additional *dynamic* calibration is performed, during which the subject rotates his/her limbs about the proximal joints (hip and shoulder) while maintaining “stiff” ankle, knee, wrist and elbow joints, imposing the same angular velocities for all IMUs. In this way, the relative orientation of the IMUs with respect to the limb can be identified, and the joint angles can then be estimated from the IMU signals. Another method for reducing integration errors caused by drift is to combine the data recorded by a three-dimensional accelerometer, a three-dimensional gyroscope and a three-dimensional magnetometer with a Kalman filter (Zhu and Zhou, 2004). The Kalman filter estimates the state parameters derived from the fusion of the three sensors by incorporating the stable drift-free performance of gravity acceleration and magnetic field (for more explanation on Kalman filtering techniques, please see Brookner, 1998). These recommendations offer the possibility of investigating other aspects of swimming locomotion, such as instantaneous velocity and/or limb orientation and its variability. According to Dadashi et al. (2016), the characterization and determination of variability in front-crawl technique descriptors may be used to distinguish swimmers’ levels, based on their skills. These authors found that skilled swimmers presented robust kinematics in response to sudden movement outbursts (i.e., a constraint manipulation). The skilled swimmers also had more diverse motor solutions than recreational swimmers, showing adaptability to surrounding constraints (e.g., velocity, fatigue, drag variations; Dadashi et al., 2016).

## Review Summary

Assessing coordination dynamics and its variability in swimming behavior may involve low-order to higher-order parameters. Investigating these parameters sheds light on the circumstances in which a swimmer exhibits behavioral stability, loss of stability or flexibility, depending on the constraints he/she is currently coping with. The swimmer–aquatic environment system has long been evaluated with video-based systems that are too limited for investigations of coordination dynamics and inter-cyclic variability. We thus show the interest of using IMUs for this type of research and underline their major advantage: they can record *continuous data over long periods*. It should nevertheless be kept in mind that these devices have undergone rapid development and the results have generally been compared against those of the gold standard in small samples. Two issues therefore require further research: (i) the *generalizability* of the results to larger populations and (ii) the *automaticity* in the data processing (regularly highlighted as an advantage of IMUs). It should be noted that using IMUs may even be inferior to video analysis if the technical recommendations in the last section of the review are not scrupulously followed. Of particular interest, IMUs can be used to determine *joint angles* as the first step in investigating the functional behavior of athletes in ecological conditions. The essential computational steps, however, cannot be ignored, from sensor placement to the correction of drift, both being critical steps in the procedure. By following the recommended procedures, researchers—as well as coaches—will be able to study swimmers’ coordination patterns and their *variability* (e.g., inter-cycle). To enhance the emergence of adaptive behaviors, it is crucial to design new training situations. This might prompt new and original perspectives for assessing this adaptability of swimmers evolving in the highly resistive environment of water. The adaptability might reveal the *evolvability* and *creativity* of swimmers, who are likely to face important and unpredictable constraints during competition.

## Author Contributions

BG, AR, DC, and LS made substantial contributions to the conception or design of the work or the acquisition, analysis, or interpretation of the data. BG, AR, DC, and LS drafted the manuscript or revised it critically for important intellectual content. BG, AR, DC, and LS gave final approval of the version to be published. BG, AR, DC, and LS also agree to be accountable for all aspects of the work and ensure that any questions related to the accuracy or integrity of any part of the work are appropriately investigated and resolved.

## Conflict of Interest Statement

The authors declare that the research was conducted in the absence of any commercial or financial relationships that could be construed as a potential conflict of interest.
